# Protective Effect of Purinergic P2X7 Receptor Inhibition on Acrolein-Induced Urothelial Cell Damage

**DOI:** 10.3389/fphys.2022.885545

**Published:** 2022-04-12

**Authors:** Zhinoos Taidi, Kylie J. Mansfield, Hafiz Sana-Ur-Rehman, Kate H. Moore, Lu Liu

**Affiliations:** ^1^ School of Medical Sciences, UNSW Sydney, Sydney, NSW, Australia; ^2^ Graduate School of Medicine, University of Wollongong, Wollongong, NSW, Australia; ^3^ St George Hospital, UNSW Sydney, Sydney, NSW, Australia

**Keywords:** acrolein, urothelium, interstitial cystitis, bladder inflammation, purinergic receptors, purinergic P2X7 receptor, urothelial permeability

## Abstract

Patients undergoing chemotherapy with cyclophosphamide experience cystitis due to excretion of a toxic metabolite, acrolein. Cystitis, an inflammation of the bladder, is associated with damage to the integrity of the urothelial barrier. The purinergic P2X7 receptor (P2X7R) is increasingly recognized for its role in inflammation and cell death. P2X7R is expressed abundantly on the bladder urothelium. The aim of this study was to investigate the role of P2X7R in acrolein-induced inflammatory damage in primary cultured porcine bladder urothelial cells. Confluent urothelial cells in culture were treated with acrolein to induce damage; also, with the P2X7R selective antagonist, A804598. Cell viability assay, immunocytochemistry, and trans-epithelial electrical resistance (TEER) studies were carried out to investigate the effect of treatments on urothelial cell function. Acrolein induced a significant reduction in urothelial cell viability, which was protected by the presence of A804598 (10 µM). The urothelial barrier function, indicated by TEER values, was also significantly reduced by acrolein, whereas pre-incubation with P2X7R antagonist significantly protected the urothelial cell barrier from acrolein-induced TEER reduction. The structure of urothelial cell tight junctions was similarly impacted by acrolein treatment, showing the fragmentation of zona occludens-1 (ZO-1) immunoreactivity. Pre-treatment of cells with A804598 countered against the actions of acrolein and maintained ZO-1 expression level and cell structure. The damaging effect of acrolein on urothelial cells integrity could be impaired by inhibition of P2X7R, therefore P2X7R blockade may be a possible therapy in patients with bladder cystitis caused by cyclophosphamide treatment.

## Introduction

Cyclophosphamide (CYP) and its toxic urinary metabolite, acrolein, causes severe cystitis, inflammation of the bladder, in patients who undergo chemotherapy. CYP-induced cystitis is one of the most frequently used models to study inflammatory cystitis (Birder and Andersson, 2018) as it displays many features similar to conditions such as interstitial cystitis/bladder pain syndrome (IC/BPS). In patients with IC/BPS, inflammation leads to the loss of or severe damage to the urothelial layer (Liu et al., 2015), absence of tight junction proteins (Slobodov et al., 2004), and increased urothelial permeability (Graham and Chai, 2006). These changes are associated with increased production of inflammatory mediators (Lamale et al., 2006; Offiah et al., 2016), histamine (Sant and Theoharides, 1994; Rudick et al., 2008), and nitric oxide (Koskela et al., 2008), as well as infiltration of inflammatory cells such as neutrophils and eosinophils (Dodd and Tello, 1998).

During bladder filling, ATP released from the urothelium is believed to function as a signaling transmitter acting on purinergic P2X2 and P2X3 receptors located on bladder afferent nerves to facilitate the sensation of fullness (Burnstock, 1972). ATP released from urothelial cells has also been shown to be increased in patients with IC/BPS (Sun and Chai, 2006) and feline model of IC/BPS (Birder et al., 2003) as well as in CYP-induced cystitis in rats (Smith et al., 2005). Patients with IC/BPS experience enhanced pain on bladder filling, which is associated with enhanced ATP release and upregulated P2X2 and P2X3 receptor expression (Tempest et al., 2004). In addition to P2X2 and P2X3 receptors, the P2X7 receptor (P2X7R) is likely to play an important regulatory role in bladder inflammatory responses seen in cystitis (Taidi et al., 2019). P2X7R expression has been shown in different layers of the urinary bladder with P2X7R immunoreactivity abundant on both the urothelium and smooth muscle (Vial and Evans, 2000; Menzies et al., 2003; Svennersten et al., 2015). Upregulation of submucosal P2X7R was detected in a mouse model of CYP-induced cystitis. In this model, treatment with a P2X7R selective antagonist significantly reduced cystitis symptoms (Martins et al., 2012).

Sustained activation of P2X7R by high concentrations of extracellular ATP (>100 µM) is associated with apoptotic cell death (Li et al., 2014), triggered by the formation of large non-selective membrane pores. These pores allow larger molecules to pass through cells membranes (Karasawa et al., 2017), including the release of apoptotic factors to promote caspase activation, and cell death (Burnstock and Knight, 2018). Previous studies have shown that substantially enhanced ATP release in response to acrolein was seen in primary rat urothelial cells and human urothelial cell lines (RT4 and T24) under basal and stretched conditions (Nirmal et al., 2014; Mills et al., 2019), indicating that increased purinergic signalling may be associated with CYP-induced cystitis and pain. However, the role of P2X7R in terms of urothelial barrier integrity and permeability in cystitis has not been explored.

Using an *ex-vivo* porcine bladder model, we have recently reported that instillation of acrolein into the bladder lumen caused damage to the urothelial and suburothelial layers, along with diminished bladder contractility in response to acetylcholine stimulation. The addition of P2X7R antagonist significantly attenuated acrolein-induced damage to the bladder (Taidi et al., 2021). The aim of this study was to further investigate whether the P2X7R is involved in acrolein-induced changes in urothelial barrier structure and integrity at the cellular level. Using primary cultured porcine urothelial cells, we have studied the role of P2X7R in acrolein-induced changes in cell morphology and viability as well as the expression of tight junction protein, zona occludens-1 (ZO-1). We have also investigated the role of P2X7R in acrolein induced disturbance of urothelial monolayer integrity by measuring trans-epithelial electrical resistance (TEER), using our newly developed *in vitro* urothelial barrier model.

## Materials and Methods

### Animal Samples

Adult female porcine bladders were harvested directly from a local abattoir and transported to the laboratory within 3 h. Upon receipt, bladders were rinsed twice with carbogenated Krebs-Henseleit solution (in mM, NaCl 118, KCl 4.7, NaHCO_3_ 25, KH_2_PO_4_ 1.2, MgSO_4_ 1.2, CaCl_2_ 2.5, and D-glucose 11.7, pH 7.4), supplemented with 1% of antibiotic-antimitotic solution (10,000 units/ml of penicillin, 10,000 μg/ml of streptomycin, and 25 μg/ml of amphotericin B Gibco, Cat# 15240062).

### Primary Porcine Urothelial Cell Isolation and Culture

The urothelial cells were scraped off from the luminal surface of the porcine bladder using a scalpel blade and suspended in RPMI 1640 culture media (R5886, Sigma-Aldrich) including 10% foetal bovine serum (FBS) (10099141, Thermo Fisher Scientific) and 1% antibiotic-antimitotic, as reported previously (Bahadory et al., 2013). Trypsin-EDTA (0.25%, 25200072, Thermo Fisher Scientific) was added to the luminal side of the bladder to remove any residual urothelial cells. After 5 min incubation at 37°C, cells were scraped off into RPMI complete media and centrifuged at 750 g for 5 min. Urothelial cells were then plated into T75 flasks and incubated at 37°C in 5% CO_2_ until they reach 70–80% confluence (approximately 7–10 days).

### Cell Morphology and Viability

Once cells achieved 80% confluence, primary urothelial cells were passaged and plated (1 × 10^5^) in a 24-well plate (Corning) and treated overnight at 37°C in 5% CO_2_ with acrolein (12.5–100 μM, 110221, Sigma-Aldrich). Cells were also treated with acrolein (50 µM) plus P2X7R antagonist, A804598 (at 1 and 10 μM, 01617, Sigma-Aldrich). Cells were monitored for changes in morphology and pictures captured at 2 h post treatment as well as after overnight treatment. Following overnight treatment, cell viability was measured using resazurin (10%, 0.3 mg/ml, ab129732, Abcam) (Riss et al., 2016; Mills et al., 2019). The fluorescence signal was quantified by Fluostar plate reader (560 nm excitation/590 nm emission) at 3 h.

### Development of *in vitro* Urothelial Barrier Model

To establish a reliable cell culture system for urothelial monolayer, we attempted to plate urothelial cells in different culture media. With some culture conditions, cells did not form high integrity barriers, or TEER values were too high to be disrupted by the application of TNFα and IL-β (both 100 ng/ml), which was used as a positive control in this study as these have been shown to markedly reduce TEER values in Caco-2 cells (Diezmos et al., 2018). A reliable system was eventually established using the following conditions: primary porcine urothelial cells were isolated and cultured in T75 flasks containing RPMI complete media until 70–80% confluence, as described above. Confluent urothelial cells were passaged and plated in permeable 12-well transwell inserts (Corning 3,413, 6.5 mm diameter inserts and 0.4 μm pore size) at 1 × 10^5^ cells per insert and incubated at 37°C in 5% CO_2_. DMEM culture media supplemented with 10% FBS, 1% antibiotic-antimitotic, and 1% glucose was used to replace RPMI. For each well, DMEM was added to both the apical (into transwell) and basal (into the well outside of the transwell) chamber. TEER or the electrical resistance of urothelial cells was measured using EVOM epithelial volt/ohm meter (EVOM, World precision instruments). TEER was monitored daily until the steady level was reached (achieved after 8–10 days in culture). The TEER value for urothelial cells was 7,390 ± 495 Ωcm^2^ (mean ± SEM), which was higher than that of Caco-2 cells (925–2,500 Ωcm^2^).

### *In vitro* Model of Damaged Urothelial Monolayer by Acrolein

Once consistent TEER values at the desired level had been reached, acrolein (at 50 µM) was applied to the apical surface of the urothelial cell layer, to induce urothelial cell damage. Application to the apical surface was chosen to mimic the *in vivo* cytotoxic effects of acrolein present in the urine of patients treated with CYP (Takamoto et al., 2004; Haldar et al., 2014). To determine the effect of P2X7R antagonist, A804598 (10 µM) on acrolein-induced urothelial cell damage, the antagonist was added to both the apical and basal cell surfaces. The antagonist applied to only the apical side had also been tried, but the results were less optimal. Urothelial cells were pre-incubated with A804598 for 30 min prior to the addition of acrolein. Baseline TEER was measured at 0 h, then changes in TEER were followed at 2, 4, 24, and 48 h after treatment.

### Immunofluorescence Staining

Immunocytochemical studies were conducted on porcine urothelial cells cultured on a glass coverslip in RPMI for 7–10 days. Confluent cells were fixed with 95% ethanol and 5% acetic acid solution for 10 min. Before use, cells were washed (3 × 10 min) with phosphate buffered saline (PBS 0.1 M, pH 7.4) followed by 30 min incubation in 10% goat or donkey serum to block nonspecific binding sites of a secondary antibody. To confirm that the primary cultured cells were of epithelial origin, cells were labelled with the cytokeratin marker AE1/AE3 antibody (1:200, M3515, Dako). To demonstrate the expression of P2X7R on isolated porcine urothelial cells, the cultured cells were incubated with primary P2X7R antibody (1:100, ab93354, Abcam) overnight at room temperature. Urothelial cell tight-junction proteins were labelled with anti-ZO-1 antibody (Invitrogen 61–7,300, 1:100) overnight at room temperature. Following incubation with primary antibodies, cells were washed (3 × 10 min) in tris-buffered saline (TBS) and tagged with a secondary fluorescent antibody for 1 h at room temperature [Alexa Fluor 594 (1:200, ab150080) for ZO-1; Alexa Fluor 488 (1:200, ab150105) for AE1/AE3, and Alexa Fluor 488 (1:200, ab150129) for P2X7R, all from Abcam]. Cells were then washed again with TBS (3 × 10 min) before the urothelial cell nuclei were stained with DAPI. Immunoreactive images were captured using Neurolucida microscope, 20/×40 objectives, and analysed using ImageJ software.

To determine the effect of the acrolein on ZO-1 expression, urothelial cells were incubated with 50 µM acrolein for 2 and 24 h in RPMI (as above) at 37°C in 5% CO_2_ before being fixed for ZO-1 immunohistochemistry. To determine the effect of P2X7R selective antagonist A804598 on acrolein-induced changes in ZO-1 tight junction protein expression, urothelial cells were pre-incubated with A804598 (10 μM) for 30 min prior to the addition of acrolein.

### Statistical Analysis

Data were analysed with GraphPad Prism 8 and expressed as mean ± standard error of the mean. One-way ANOVA followed by Bonferroni’s multiple comparisons was applied to analyse cell viability results, and two-way ANOVA followed by Bonferroni’s multiple comparisons was used to analyse the TEER results. *p* value <0.05 was set as statistical significance.

## Results

### AE1/AE3 and P2X7R Immunofluorescence Staining

**FIGURE 1:**
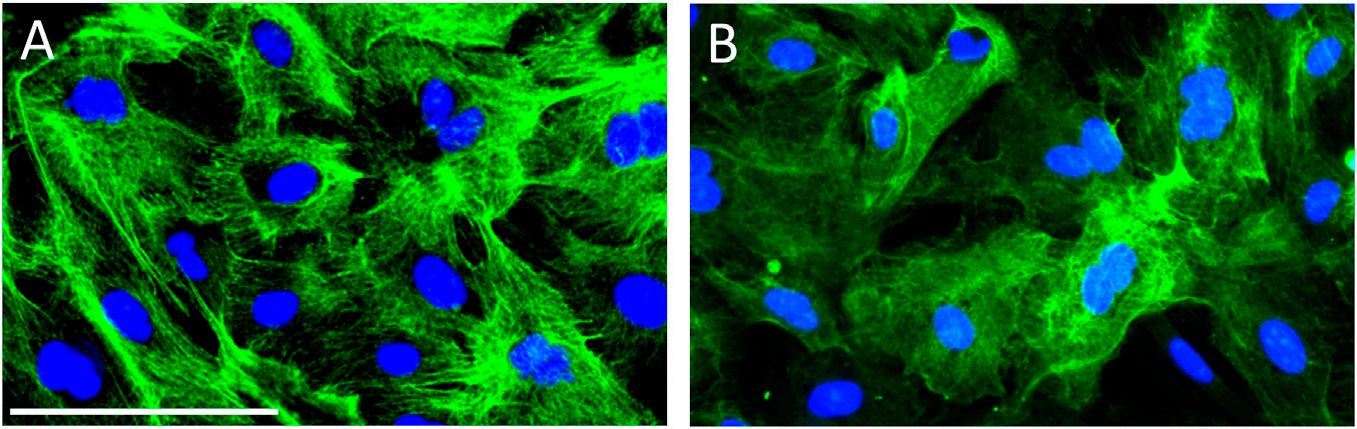
Immunocytochemistry on porcine primary cultured urothelial cells. **(A)** All cultured cells uniformly expressed AE1/AE3 cytokeratin, the epithelial cell marker, indicating the pure identity of cultured porcine urothelial cells. **(B)** Cultured urothelial cells show an intense expression of P2X7R in the cytoplasm and membranes. The green color denotes AE/AE3 staining in panel A and P2X7R staining in panel B. The blue color shows the nuclei stained with DAPI. Magnification bar shows 50 µm.

As shown in Figure 1A, all cultured porcine urothelial cells were positive immunoreactivity against AE1/AE3, verifying the epithelial origin of these cells. P2X7R immunoreactivity was demonstrated in the cytoplasm and membranes of most but not all primary cultured porcine urothelial cells (Figure 1B), confirming the expression of P2X7R on cultured urothelial cells.

### Effect of P2X7R Inhibition on Acrolein-Induced Cytotoxicity

**FIGURE 2:**
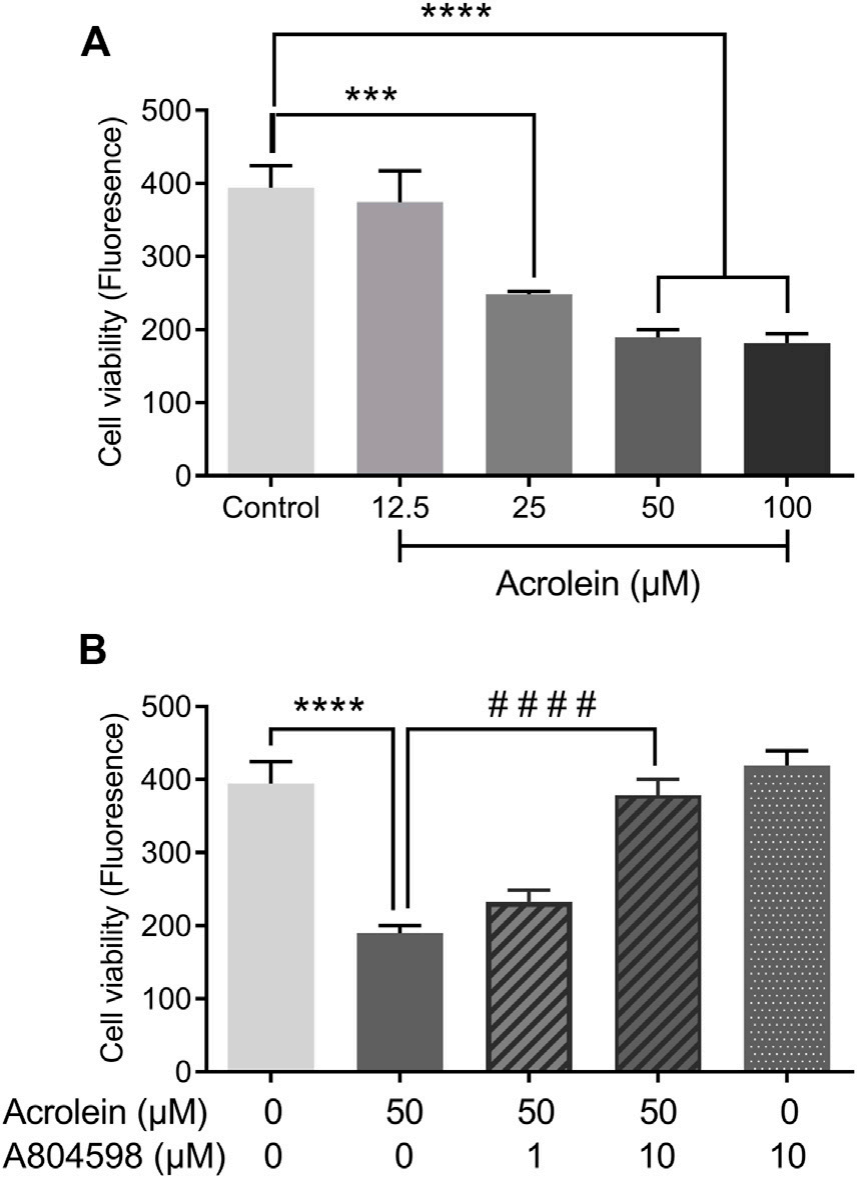
Effects of P2X7R inhibition on acrolein-induced cytotoxicity as measured by oxidation of resazurin. **(A)** Acrolein reduced urothelial cell viability in a concentration-dependent manner (12.5–100 µM). **(B)** The reduced cell viability caused by acrolein (50 µM) was prevented by the addition of P2X7R antagonist A804598 at 10 μM, although the preventive effect was not significant at a lower concentration (1 µM). A804598 (10 µM) alone showed no different compared to the control. ***p < 0.001, ****p < 0.0001 compared to the control; ^####^ *p* < 0.0001 compared to the acrolein treated group (*n* = 6 for all groups, one-way ANOVA followed by Bonferroni’s multiple comparisons.

Acrolein markedly reduced the urothelial cell viability in a concentration-dependent manner (Figure 2A). As the concentration of acrolein increased to 25 and 50 µM there was an increasing cytotoxic effect seen by the reduction in cell viability (*p* < 0.0001). This appeared to plateau at 50 µM acrolein. Therefore, the effect of the P2X7R selective antagonist (A804598) was examined on cells treated with 50 μM acrolein (Figure 2B). The cytotoxic effect of acrolein was not significantly inhibited by the pre-treatment of cells with A804598 at 1 µM A804598 at 10 μM, however, showed a great impact on the cytotoxic effect of acrolein and significantly abolished the acrolein induced cytotoxicity (*p* < 0.0001). The result of A804598 alone was similar to that of the control group.

### Effect of P2X7R Inhibition on Acrolein-Induced Changes in Cell Morphology

**FIGURE 3:**
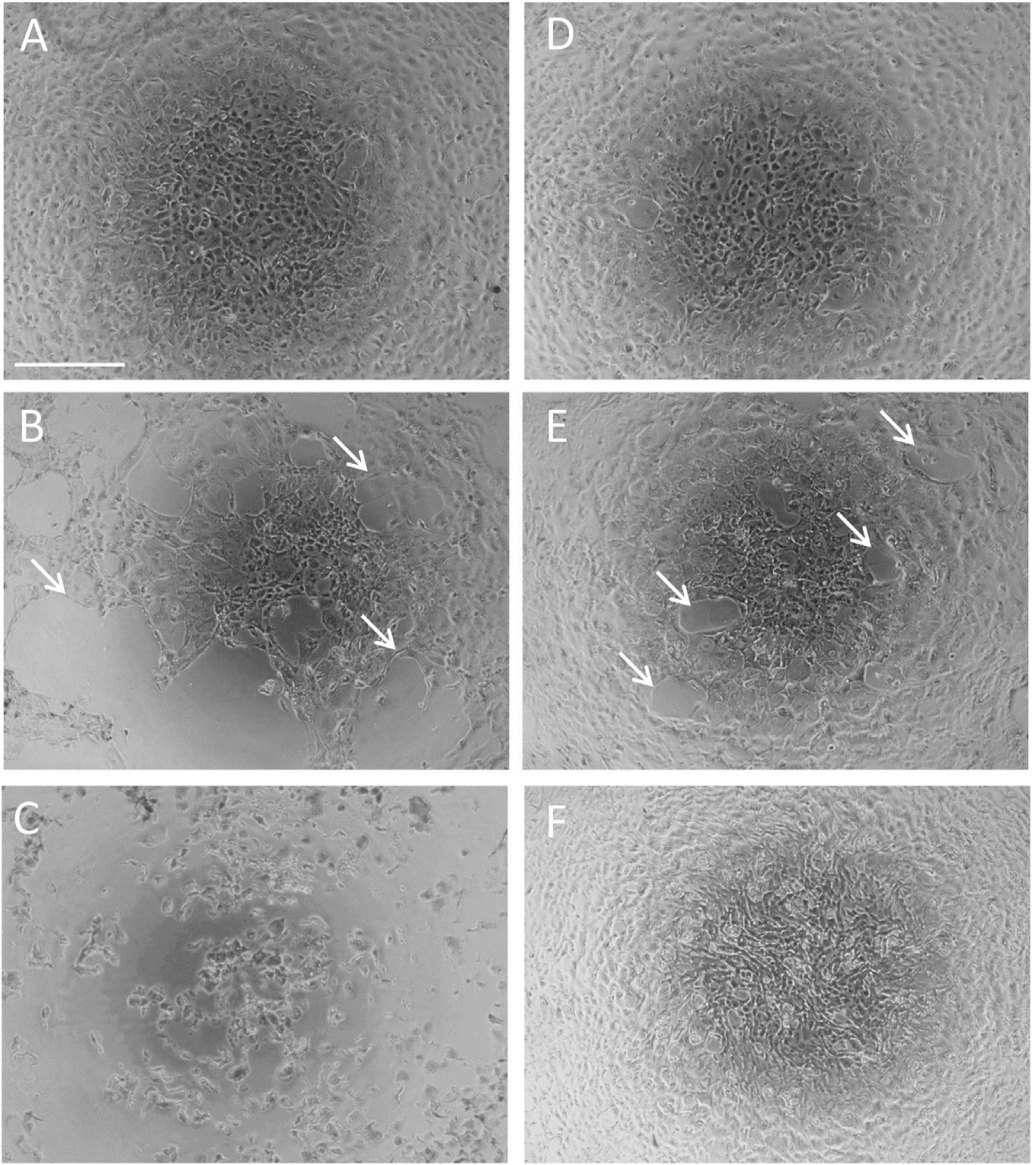
Effect of P2X7R inhibition on acrolein-induced changes in cell morphology. Confluent primary cultured urothelial cells under normal culture conditions **(A)**. Cells were treated with acrolein (50 µM) for 2 h **(B)** or overnight **(C)**. Cells were treated with the selective P2X7R antagonist A804598 (10 µM) overnight **(D)**. Cells were pre-incubated with A804598 (10 µM) for half an hour and then treated with acrolein (50 µM) for 2 h **(E)** or overnight **(F)**. Magnification bar shows 100 µm. White arrows indicate the disruption of cell-cell adhesions.

Urothelial cells freshly isolated from the luminal surface of the porcine urinary bladder were initially rounded in shape. After three to 4 days in culture, cells flattened and spread out. After 7–10 days in culture, cells appeared confluent with urothelial cells forming a well-connected monolayer of epithelial-like shaped cells with a typical cobble-stone morphology (Le et al., 2014) (Figure 3A).

Treatment of the cultured urothelial cells with 50 µM acrolein for 2 h resulted in obvious damage to the urothelial cell monolayer. Attached and well-connected urothelial cells became dissociated at several points within the monolayer (indicated by white arrows in Figure 3B). Further incubation overnight with 50 µM acrolein enhanced this dissociation, with cells losing their flattened, cobble-stone morphology, and becoming spherical or irregular in shape with more cells losing adherence, indicative of an increase in dead cells (Figure 3C). Incubation of the urothelial cells with the selective P2X7R antagonist, A804598 (10 µM) by itself, did not cause any changes in cell morphology (Figure 3D). Pre-incubation of urothelial cells with A804598 (10 µM) protected the urothelial monolayer from the disruptive effects of acrolein at 2 h (Figure 3E) and overnight (Figure 3F). Although minor dissociation was still seen on urothelial monolayer after 2 h incubation with acrolein (50 µM) following pre-treatment with A804598 (Figure 3E, white arrows), cells appeared to proliferate overnight and return to a normal cobble-stone morphology (Figure 3F).

### Effect of P2X7R Inhibition on Acrolein-Induced Disruption to Urothelial Cell Integrity

**FIGURE 4:**
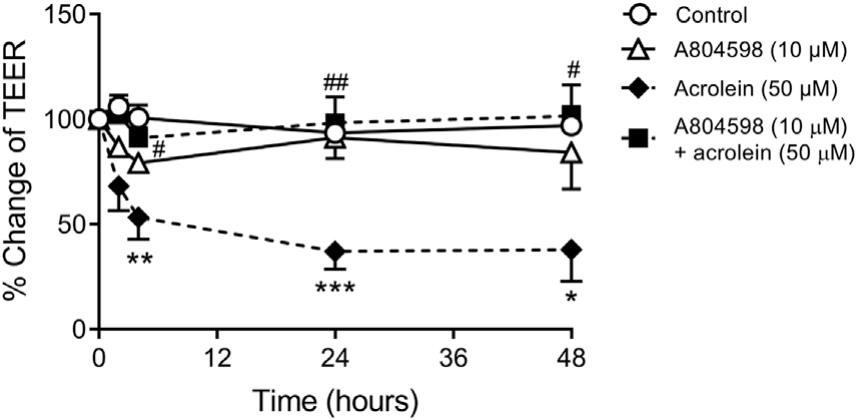
Effect of P2X7R inhibition on acrolein-induced disruption to urothelial cell integrity measured by TEER. Data were expressed as the percentage change of TEER relative to pre-treatment of the same well. Two-way ANOVA analysis showed a highly significant reduction in TEER after urothelial cells were treated with acrolein (50 µM) up to 48 h (****p < 0.0001 compared to control). Acrolein induced TEER reduction was significantly prevented by co-incubation with P2X7R antagonist A804598 (10 µM) (^#####^ *p* < 0.0001 compared to acrolein alone). A804598 alone did not show any effect on TEER values. Each condition was measured as duplicates of *n* = 8 animals. Two-way ANOVA followed by Bonferroni’s multiple comparison tests show *p < 0.05, **p < 0.01, ***p < 0.001 compared to the same time point of the control group; ^#^ *p* < 0.05, ^##^ *p* < 0.01 compared to the same time point of the acrolein alone group.

Treatment of cultured porcine urothelial cells with 50 µM acrolein caused significant disruption to urothelial cell integrity as indicated by a decrease in TEER values (Figure 4). After 24 h of acrolein treatment, there was a 60% reduction in trans-epithelial resistance. Pre-incubation of urothelial cells with the P2X7R antagonist A804598 (10 µM) showed protection against acrolein-induced disruption to urothelial cell integrity, as the TEER values returned to the control level, which was significantly different from that of the acrolein only group between 4 and 48 h. A804598 by itself did not lead to any significant changes in TEER compared to control.

### Effect of P2X7R Inhibition on Acrolein-Induced Disruption to Urothelial Cell Tight Junction Protein ZO-1

**FIGURE 5:**
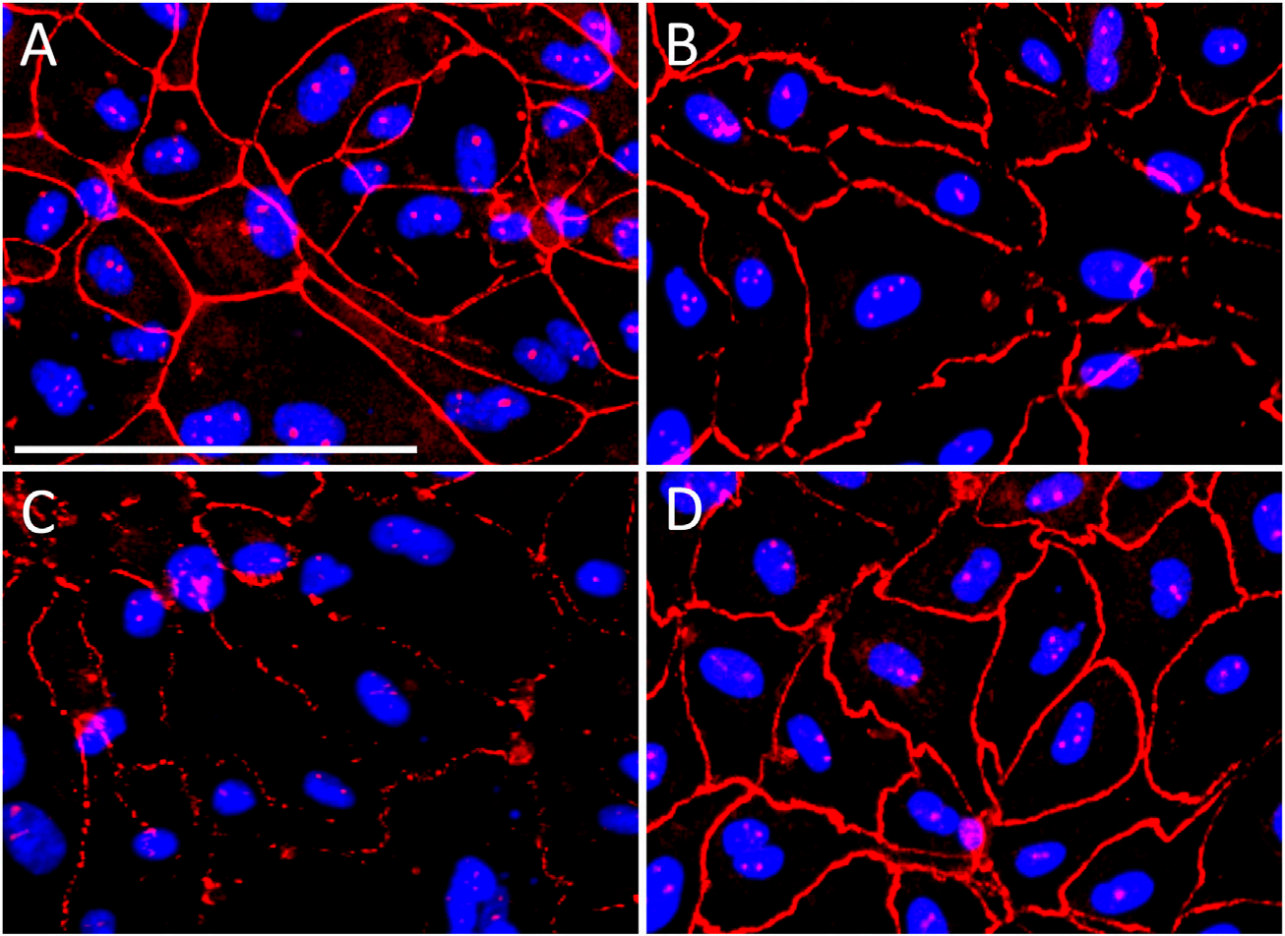
Immunocytochemistry of tight junction protein zonula occludens-1 (ZO-1) (red) on primary cultured porcine bladder urothelial cells. ZO-1 immunoreactivity remained intact in **(A)** control cells and **(B)** cells treated with P2X7R antagonist A804598 (10 µM). Cells treated with acrolein (50 µM) for 4 h disrupted the integrity of ZO-1 immunoreactivity **(C)**. ZO-1 immunoreactivity remained intact in acrolein treated cells in the presence of A804598 (10 µM) **(D)**. The blue color shows the nuclei stained with DAPI. Magnification bar shows 100 µm.

The integrity of the urothelial cell monolayers formed after 8–10 days in culture was demonstrated by strong immunoreactivity of tight junction protein ZO-1 (Figure 5A). Acrolein induced cytotoxicity and disruption to the urothelial cell integrity was demonstrated by the reduction in ZO-1 immunoreactivity. ZO-1 immunoreactivity was clearly discontinuous and highly compromised in urothelial cells treated with acrolein (50 µM) for 4 h (Figure 5C) compared to controls. However, when cells were treated with both A804598 (10 μM) and acrolein (50 μM), the disruption to ZO-1 expression was inhibited (Figure 5D), indicating that A804598 protected urothelial cell monolayers from acrolein-induced damage. A804598 (10 μM) alone did not show any effect on ZO-1 expression (Figure 5B).

## Discussion

Interstitial cystitis and other inflammatory bladder conditions are associated with changes in the urothelial cell viability, integrity, and permeability. The current study has demonstrated that urothelial cell changes induced by acrolein are very similar to those demonstrated in patients with bladder conditions associated with IC/PBS. Importantly, this study has shown that these changes can be inhibited by the selective P2X7R antagonist, A804598.

The urothelium of the bladder from patients with IC/BPS is thinner, usually only 1 or 2 cells thick, compared to the urothelium from normal bladders, which is approximately 5 cells thick (Slobodov et al., 2004). Decreased urothelial cell viability and increased apoptotic cell death are common in cystitis. They have been demonstrated in IC/BPS (Shie and Kuo, 2011; Shie et al., 2012) and other type of urinary bladder cystitis including ketamine-induced cystitis (Lee et al., 2013). Increased urothelial cell apoptosis is also common in other bladder conditions, including bladder outlet obstruction, spinal cord injury, and recurrent urinary tract infection (Liu et al., 2015). As supported by the findings in the current study, acrolein is a well-known cytotoxic compound with effects on cell viability reported for doses between 5 µM (Suabjakyong et al., 2015) and 100 µM (Mills et al., 2019).

As well as decreases in urothelial cell viability, cystitis is associated with disruption to the integrity of the urothelial barrier. Absence of ZO-1 (Slobodov et al., 2004) and increased urothelial permeability (Graham and Chai, 2006) have been reported in patients with haemorrhagic cystitis and also in animal models of CYP-induced cystitis or intravesical instillation of acrolein (Martins et al., 2012). Similar findings have been reported in other tissues with decreased epithelial integrity and increased epithelial barrier permeability observed in *in-vitro* models of acrolein-treated vocal fold epithelial cells (Liu et al., 2016) and intestinal epithelial cells (Chen et al., 2017). The present study demonstrated the expression of tight junction protein, ZO-1, in cultured porcine urothelial cells and that acrolein treatment has led to the degradation of tight junction protein. Increased urothelial cells permeability as a result of damaged or lost tight junction proteins will eventually cause the pathophysiological condition in which normal cell homeostasis is disturbed. In the current study this was demonstrated by a decrease in trans-epithelial resistance, indicating a disruption of the normal barrier functions of the urothelium.

Perhaps the most important finding of the current study was that the acrolein-induced changes in urothelial cell viability, integrity, and permeability were significantly inhibited by pre-treatment of urothelial cells with the selective P2X7R antagonist, A804598. This is the first report of the protective effect of P2X7R inhibition on acrolein-induced changes in urothelial cell permeability, although similar and comparable results have been reported previously in other models examining epithelial barrier integrity (Chen et al., 2014; Wu et al., 2017). This study is also the first to report the protective effect of A804598 on the expression of tight junction proteins in primary urothelial cells, which is in line with previous studies reported in other systems. For example, brilliant blue G, which is a selective non-competitive P2X7R antagonist, preserved the expression of both occludin and ZO-1 in the rat lung tissue in a model of neurogenic pulmonary edema (Chen et al., 2014). Also, in the model of sepsis-induced intestinal barrier disruption (Wu et al., 2017), P2X7R antagonist not only increased TEER but also showed a significant increase in expression of tight junction proteins, occludin, claudine-1, and ZO-1 (Wu et al., 2017). The same study showed that Bz-ATP (a purinergic agonist with potency for P2X7R) significantly decreased the expression of tight junction proteins and reduced TEER in the intestine (Wu et al., 2017).

The P2X7R antagonist, A804598, also protected against acrolein induced loss of cell viability. The effects of P2X7R on cell viability appears less clear cut in the literature. Although it is generally well received that P2X7R activation can cause apoptosis/necrosis and cell death due to the formation of large cytoplasmic pores, resulting in increased cell permeability (Schulze-Lohoff et al., 1998), there are reports showing that activation of P2X7R increases cell proliferation in lymphoid cells (Baricordi et al., 1999) and microglia cells (Bianco et al., 2006). The opposing roles of P2X7R in both cell proliferation and cell death are proposed to be dependent on the concentration of extracellular ATP. It is hypothesised that low concentrations of extracellular ATP lead to basal activity of P2X7R, which results in increased cell proliferation (Adinolfi et al., 2005); however, high extracellular ATP levels lead to apoptosis and cell death (Haanes et al., 2012). As mentioned previously, interstitial cystitis is associated with increased ATP release from urothelial cells (Sun and Chai, 2006). Therefore, in these conditions, blocking the P2X7R can protect urothelial cells from death. The second important consideration is that the P2X7R itself is an important regulator of extracellular ATP level (Brandao-Burch et al., 2012), leading to ATP-induced ATP release, via P2X7R and other ion channels, such as pannexin channels (Dahl, 2015; Ceriani et al., 2016). Therefore, P2X7R antagonism will not only inhibit urothelial cell apoptosis in the presence of high concentrations of extracellular ATP it will also prevent excessive ATP release into the extracellular space.

Taken together, the results of the current study indicate that acrolein decreases urothelial cell viability and disrupts the urothelial barrier integrity by damaging tight junction proteins, leading to increased urothelial barrier permeability. These changes, which are analogous to the changes seen in patients with interstitial cystitis, were inhibited by pre-treatment of urothelial cells with P2X7R antagonist, A804598. These findings are in line with our previously reported observations in our *ex-vivo* model of urothelial damage by direct instillation of acrolein into the whole porcine bladder lumen (Taidi et al., 2021) and provide further evidence for the potential of P2X7R antagonists in the treatment of IC/PBS.

Recently, many researchers have investigated the effect of P2X7R antagonists on lower urinary tract inflammatory diseases (Goncalves et al., 2006; Martins et al., 2012; Munoz et al., 2017). Martins and associates reported a decrease in the pain response in the CYP-treated animals following treatment with a P2X7R antagonist or genetic removal of P2X7R (Martins et al., 2012). They showed that the treatments result in decreased bladder inflammatory responses as well as reduced edema and hemorrhage (Martins et al., 2012). Another study has investigated the possible role of P2X7R in inflammatory pain in kidneys with unilateral ureteral obstruction. In P2X7R knockout mice, there was a reduction in inflammatory cell infiltration and a reduction in tubular apoptosis (Goncalves et al., 2006). Also, in a similar model of acute ischemic kidney injury in mice, the P2X7R antagonist A438079 reduced the expression of inflammatory chemokines (Yan et al., 2015). Likewise, in a murine colitis model, treatment with a P2X7R antagonist, A438079, reduced the production of inflammatory cytokines, TNF and IL-1β, in colon tissue (Wan et al., 2016). P2X7R antagonists have advanced to clinical trials as a treatment for a number of inflammatory diseases, including rheumatoid arthritis and inflammatory bowel disease (Park and Kim, 2017). The P2X7 antagonist AZD9056 has shown positive results in phase IIa trials, with improvement in pain related symptoms in patients with Crohn’s disease (Eser et al., 2015).

Overall, the results of the current study have provided evidence for the therapeutic potential of P2X7R antagonists for inflammatory bladder diseases. Inhibition of P2X7R activity could be a pathway for the treatment of bladder inflammation and could potentially be co-administered with CYP in patients undergoing chemotherapy, thereby protecting them against treatment associated cystitis. Further studies should be conducted to investigate the effectiveness of different P2X7R antagonists. Our *in-vitro* barrier model developed using porcine urothelial cells can also be used to evaluate the effect of current treatments of IC/BPS on urothelial TEER, tight junction formation and viability to compare their efficacy in protecting urothelial integrity.

## Data Availability

The raw data supporting the conclusion of this article will be made available by the authors, without undue reservation.

## Abbreviations

CYP, cyclophosphamide; IC/BPS, interstitial cystitis/bladder pain syndrome; P2X7R, P2X7 purinergic receptor; TEER, trans-epithelial electrical resistance; ZO-1, zona occludens-1.
